# Consultation and citation rates for prior imaging studies and documents in radiology

**DOI:** 10.1117/1.JMI.5.3.031409

**Published:** 2018-05-08

**Authors:** Tamara Miner Haygood, Barry Mullins, Jia Sun, Behrang Amini, Priya Bhosale, Hyunseon C. Kang, Tara Sagebiel, Bilal Mujtaba

**Affiliations:** a University of Texas MD Anderson Cancer Center, Department of Diagnostic Radiology, Houston, Texas, United States; b University of Texas MD Anderson Cancer Center, Department of Biostatistics, Houston, Texas, United States

**Keywords:** radiology report citation rates, radiology old reports, radiology documentation

## Abstract

Frequently, the consensus conclusion after quality assurance conferences in radiology is that whatever mistake was made could have been avoided if more prior images or documents had been consulted. It is generally assumed that anything that was not specifically cited in the report had not been consulted. Is it actually safe to assume that an image or document that is not cited was also not consulted? It is this question that this investigation addresses. In this Institutional Review Board-approved study, one observer watched the board-certified radiologists while they interpreted imaging studies and issued reports. He recorded what type of study was being interpreted [either computed tomography, magnetic resonance imaging, or conventional radiography (x-ray)]. He also recorded the number and type of prior imaging studies and documents that were consulted during the interpretation. These observations were then compared with the signed report to determine how many of the consulted imaging studies and documents were cited. Of the 198 previous imaging studies that the radiologists consulted, 116 (58.6%) were cited in a report. Of the 285 documents consulted, 3 (1.1%) were cited in a report. This difference in citation rate was statistically significant (p<0.0001). It cannot be safely assumed that an older radiologic image or medical document was not consulted during radiologic interpretation merely because it is not cited in the report. Radiologists often consult more old studies than they cite, and they do not cite the majority of prior documents that they consult.

## 1 Introduction

In many quality assurance meetings in radiology, the consensus conclusion is that a mistake could have been avoided if more prior images or documents had been consulted. It is assumed that any previous study or document that was not specifically cited in the report was not consulted. We sought to test the validity of this assumption. Is it safe to assume that an image or document that is not cited was also not consulted? This is the question that this investigation addresses.

Although there are some dissenting opinions, particularly regarding the need to compare an apparently normal examination with prior studies, the consensus opinion in the radiological literature seems to be that comparison with older studies is helpful.^1^^,^^2^

There is little available in the literature concerning use of written documents in formulating reports on current imaging studies. Most of this has focused on the use of the radiology report. Kevin Berbaum and colleagues found that both the old images and the old reports were useful in helping radiologists reach a more specific conclusion from the images being interpreted and in increasing their confidence in that interpretation, but the old images were more helpful than the reports, with the old report being used mainly to provide patient history or the previous radiologist’s opinion.^3^^–^^5^ There can be a downside to conferring with the prior reports as it can lead to alliterative errors, in which the reporting radiologist follows a previous colleague down an incorrect path.^2^

There has been even less attention devoted to comparison with old documents other than the radiology report. The American College of Radiology, in a practice parameter updated in 2014, stated that, “Comparison with relevant examinations and reports should be part of the radiologic consultation and report when appropriate and available.”^6^^,^^7^

It did not specifically say whether only reports of previous imaging studies were intended or whether the suggestion would extend to other written documents, and it is also not clear whether an imaging report should be cited separately or whether the report of a radiological study might be considered to go along with citation of the actual imaging study.

We believe that prior imaging studies and imaging reports and other documents can be useful in generating a helpful and accurate report of the current imaging study. Pathology reports, for example, can let the radiologist know what disease the patient has and access to a radiation-therapy planning image can confirm that abnormal signal in soft tissues on magnetic resonance imaging (MRI) is due to radiation exposure. We sought to document patterns of usage and citation of previous images and documents by our radiologists.

## 2 Methods

Radiologists were observed by one investigator over several sessions ranging from 1 to 3 h in length during April or May 2015. These reading sessions occurred during the reader’s scheduled clinical time. The reader chose computed tomography studies (CTs), radiographs, and/or MRI studies from the queue of available studies and interpreted them. This was real-time, prospective, clinical reading of current studies without any changes to normal workflow. The investigator positioned himself so he could see the monitors on which the radiologist was conducting the interpretation and recorded observations using pencil and paper. We included only interpretations of studies that had, at a minimum, one prior imaging study available for comparison. Interpretations were performed on workstations that integrated Philips Intellispace Picture Archiving and Communications System (PACS), Nuance PowerScribe 360, and the University of Texas MD Anderson Cancer Center Clinic Station, an internally developed electronic medical record (EMR).

For each interpretation, the investigator recorded the modality, date of observation, date of study, and medical record number of the patient whose study was being interpreted. The investigator then documented, using tally marks, the reader’s use of any available comparison CT, MRI, radiography, ultrasound, positron emission tomography, or other nuclear imaging, including any imaging performed at another institution but available via PACS at the time of interpretation. In addition, the investigator documented the reader’s use of clinical documentation, from the institution-specific EMR, in the following categories:

a. Reports from prior imaging;
b. Pathology reports;
c. Laboratory data;
d. Transcribed documents;
e. Scanned documents;
f. Radiation oncology treatment plans.

Transcribed documents included documentation from history and physical examinations, clinical notes, progress notes, consultations, operative or other procedure reports, research protocol documents, and discharge summaries. The observer did not record the specific type of transcribed document that was consulted. Scanned documents included similar types of documentation when performed at outside facilities, with paper copies scanned into the EMR. Again, the observer did not record the specific type of scanned document that was consulted.

Upon completion of a reading session, the data collection sheets were paired with a copy of the final, dictated report from the respective imaging interpretation. The patient’s medical record number was then removed from the data sheet. The report was compared with the data collection sheet to identify any reference to the comparison imaging and/or clinical documentation utilized in image interpretation.

The observing investigator then transferred the data to a spreadsheet/database. Key components of the database included a three-digit code to identify the reader but no patient identifiers, the number and types of comparison information used during image interpretation, and the number of comparison studies and/or reports cited in the final report. A second investigator then compared the data on the spreadsheet to the data collection sheets for accuracy. Upon verification of accurate transfer to the spreadsheet, the data collection sheets were destroyed.

Board-certified staff radiologists served as readers in this study. Readers knew that they would be observed in interpretation of imaging studies and that the investigators were interested in some of the things they might do while reading studies, but they did not know exactly what was being studied. Specifically, the goal of documenting their use of comparison imaging and written items of patient history was withheld. This was done purposefully, in order to observe each reader in an environment mirroring that of routine, daily workflow as closely as possible and to avoid influencing their behavior in this regard. We also obtained background information on each reader regarding specialization and an estimated quantity of studies interpreted per year.

Readers gave informed consent to participate in this Institutional Review Board-approved study. Patient consent was waived by the Institutional Review Board.

Statistical analysis was performed using a two-sided Fisher’s exact test with p-values of 0.05 or less considered statistically significant. An exception was evaluation of the proportion of consulted imaging studies that were of the same type as the study being interpreted. For that analysis, a two-sided exact binomial test was used, again with a p value of 0.05 being considered significant. Statistical analysis was carried out using SAS version 9 (SAS Institute, Cary, North Carolina, USA).

## 3 Results

Five radiologists agreed to participate. They were all certified by the American Board of Radiology and had completed residency and achieved certification between 2004 and 2012. Two specialized in musculoskeletal imaging and three in abdominal imaging. One declined to estimate numbers of imaging studies interpreted. The others estimated an average of 75 CTs interpreted per week (range 50 to 100), 19 MRIs interpreted per week (range 5 to 30), and 21 conventional radiographs (range 10 to 50).

Our five participating radiologists issued 62 reports. The reports were fairly evenly spread among the participants with each radiologist averaging ∼12 reports (range 6-24) (Table 1).

**Table 1:** Summary of consulted and cited imaging and documents.

Study type	Reports issued	Imaging studies consulted	Cited imaging studies	Percentage of imaging studies cited in the report	Documents consulted	Documents cited	Percentage of documents cited in the report
CT	44	148	86	58.1%	233	3	1.30%
MRI	7	27	16	59.3%	29	0	0%
Radiograph	11	23	14	60.9%	23	0	0%
Total	62	198	116	58.6%	285	3	1.1%
Reader number							
1	6	18	17	94.4%	22	1	4.5%
2	11	36	14	38.9%	49	0	0.0%
3	24	77	48	62.3%	87	1	1.1%
4	10	39	21	53.8%	97	0	0.0%
5	11	28	16	57.1%	30	1	3.3%
Total	62	198	116	58.6%	285	3	1.1%

While issuing these 62 reports, the radiologists consulted 198 previous imaging studies and 285 documents.

That equates to an average of 3.2 imaging studies consulted per issued report (range 0 to 8, median 3) and an average of 4.6 documents consulted per report (range 0 to 13, median 3.5). The greater frequency of consultation of documents compared with imaging studies was statistically significant (p=0.0017).

**Fig. 1:**
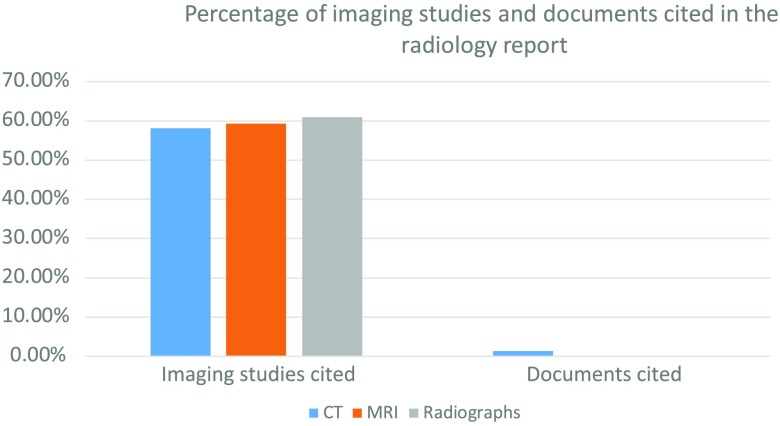
Of the 198 previous imaging studies that the radiologists consulted, 116 (58.6%) were cited in a report. Of the 285 documents consulted, 3 (1.1%) were cited in a report. This difference was statistically significant (p<0.0001). The pattern of radiologist citation and review of prior studies was similar among all of the board-certified radiologists who served as our participants. The percentage of reports that cited fewer studies than were consulted ranged from 16% to 72%. Out of 62 total reports, fewer studies were cited than consulted in 33 (53.2%).

Of the 198 previous imaging studies that the radiologists consulted, 116 (58.6%) were cited in a report. Of the 285 documents consulted, 3 (1.1%) were cited in a report. This difference in citation rate was statistically significant (p<0.0001). See Fig. 1. The pattern of radiologist citation and review of prior studies was similar among all of the board-certified radiologists who served as our participants. The percentage of reports that cited fewer studies than were consulted ranged from 16% to 72%. Out of 62 total reports, fewer studies were cited than consulted in 33 (53.2%).

The interpreting radiologists consulted a variety of documents (see Table 2).

**Table 2:** Consultation and citation of documents.

Study type	Reports issued	Document type consulted	Total number consulted	Number cited	Percentage cited
CT	44	Radiology report	92	1	1.1%
		Transcribed document	82	1	1.2%
		Scanned document	6	0	0.0%
		Radiation treatment plan	2	1	50.0%
		Pathology report	44	0	0.0%
		Laboratory report	7	0	0.0%
		Total	233	3	1.3%
MRI	7	Radiology report	10	0	0.0%
		Transcribed document	13	0	0.0%
		Pathology report	6	0	0.0%
		Total	29	0	0.0%
X-ray	11	Radiology report	2	0	0.0%
		Transcribed document	17	0	0.0%
		Pathology report	2	0	0.0%
		Laboratory report	2	0	0.0%
		Total	23	0	0.0%
		Total for all	285	3	1.1%

Radiologists tended to consult the same type of imaging study as that which they were interpreting (see Table 3).

**Table 3:** Comparison of study type interpreted and consulted.

Study type interpreted	Consulted same type as being interpreted	Total studies consulted	Percentage	95% confidence interval	p-value
CT	115	148	78%	0.70 to 0.84	<0.0001
MRI	22	27	81%	0.62 to 0.94	0.002
X-ray	13	23	57%	0.34 to 0.77	0.60

## 4 Discussion

This prospective study demonstrated that our radiologists will consult a wide variety of sources if those sources are readily available. Our radiologists used on average 3.2 comparison imaging studies and 4.6 written documents in their issuance of each report. Our radiologists then cited over half of the imaging studies that they consulted but only three of the written documents. The difference in the rate of citation was overwhelming.

Our results suggest that a radiologist cannot be assumed not to have consulted any particular prior study or document merely because it is not cited in the report. This may have implications in medicolegal contexts, for quality assurance purposes, and for other researchers. For example, Lakhani et al. used structured query language to determine how often radiologists compared with prior studies when issuing 1.8 million reports stored in a PACS database. They concluded that prior examinations were used in 38.69% of the reports. They suggested that it was possible that this percentage was an underestimate because a prior study might have been used but not mentioned, and our research indicates that this is very likely.^8^

Why were so few documents cited? Most likely the answer lies in three factors. One is a desire to keep reports to a manageable length. A second is that the fingertip accessibility of multiple types of documents is a very recent phenomenon in radiology. For decades it has been accepted practice that one should consult prior imaging studies and document that consultation in the report, and generally the old images, and sometimes the reports of those images, were readily available. Consultation with other prior resources, however, has not been a routine part of radiology practice, and neither the consultation itself nor documentation of the consultation has received much attention in the medical literature.

A third factor lies in the structure of the report. At our institution (and probably at most), comparison studies are listed near the beginning of the report. Assuming that most radiologists start at the beginning of the report and fill in information such as the patient’s name, study type, and history as well as the dates of comparison studies before taking more than a cursory glance at the images, the need or desire to consult additional prior images or documents may not arise until the interpreting radiologist is in the middle of the report. When reports are dictated on tape for a transcriptionist, it is cumbersome to add in another citation when partway through the dictation. With voice recognition, it is easier, but for many radiologists, it requires going against habits built before the introduction of voice recognition.

Our study has some limitations. We observed five radiologists, all of whom work at a tertiary care facility where patients often return over and over for years. Therefore, an abundance of older images and documents usually awaits the enterprising radiologist who may wish to consult them during interpretation of a new study. One would expect that in many other settings (the emergency department of a general hospital, for example) fewer prior studies and documents might be available and therefore fewer would be consulted.

## 5 Conclusion

It cannot be safely assumed that an older radiologic image or medical document was not consulted during radiologic interpretation merely because it is not cited in the report. Radiologists often consult more old images than they cite, and they do not cite the vast majority of prior documents that they consult.
